# Pseudohypercalcemia in Multiple Myeloma: A Case Report 

**Published:** 2017-07-01

**Authors:** Farzaneh Ashrafi, Bijan Iraj, Pardis Nematollahi, Ali Darakhshandeh

**Affiliations:** 1 Hematologist and Medical Oncologist, Isfahan University of Medical Sciences, Isfahan, Iran; 2 Endocrinologist, Isfahan University of Medical Sciences, Isfahan, Iran; 3 Pathologist, Isfahan University of Medical Sciences, Isfahan, Iran; 4 Internist, Isfahan University of Medical Sciences, Isfahan, Iran

**Keywords:** Multiple myeloma, Psudohypercalcemia

## Abstract

Hypercalcemia is a common finding in patients with multiple myeloma. Clinical manifestations of hypercalcemia correlate with the level of serum calcium. Ionized serum calcium (Ca (I)) will be increased in true hypercalcemia. In pseudohypercalcemia the Ionized Ca is normal, although binding of calcium to abnormal immunoglobulin causes increased serum calcium level. In the asymptomatic multiple myeloma patients with moderate to severe hypercalcemia, measurement of ionized calcium is critical to exclude pseudohypercalcemia. Here, we describe an asymptomatic 44-year-old man with multiple myeloma who had severe hypercalcemia, but normal serum Ionized Ca level.

## Introduction

 Hypercalcemia is a common biochemical disorder in clinical practice, which can be associated with a variety of clinical manifestation ranging from asymptomatic to life threatening conditions such as renal failure and coma. Generally, severity of clinical manifestation correlates with degree and chronicity of hypercalcemia^1^. Malignancy and primary hyperparathyroidism are the most common causes of hypercalcemia, accounting about 90% of cases. Multiple myeloma is one of the most common malignancies associated with hypercalcemia. Approximately 28% of myeloma patients have elevated serum calcium at the time of diagnosis^2^.

## CASE PRESENTATION

**Figure 1:**
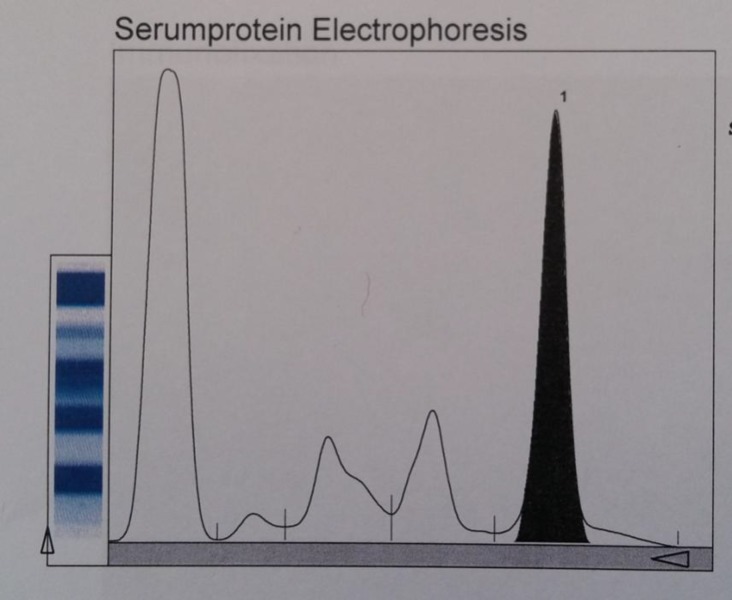
Densitometry revealing a monoclonal

A 44 – year- old man admitted to hospital because of sever hypercalcemia. He has had a history of progressive fatigue and generalized bone pain from three months ago. Other clinical signs and symptoms of clinical hypercalcemia such as renal, gastrointestinal, neurologic and cardiovascular manifestations were absent. He had no history of medical disease and was taking no medications except of analgesics for bone pain. Laboratory data showed serum calcium (Ca) 17.9 mg/dL, albumin 3.9 g/dL, phosphorous (P) 2.3 mg/dL, iPTH 19 pg/ml, ESR 45 mm/h, creatinine 0.9 mg/dL, 25 (OH)Vit D3 4 nmol/L, Hb 10.5 g/dL, hematocrit 31%. The presence of hypercalcemia, elevated ESR, anemia and low PTH raised the probability of Multiple Myeloma. Serum protein electrophoresis and immunofixation showed severe hypergammaglobulinemia (8.7 g/dL) and IgG Kappa monoclonal gammapathy (Figure 1).

**Figure 2:**
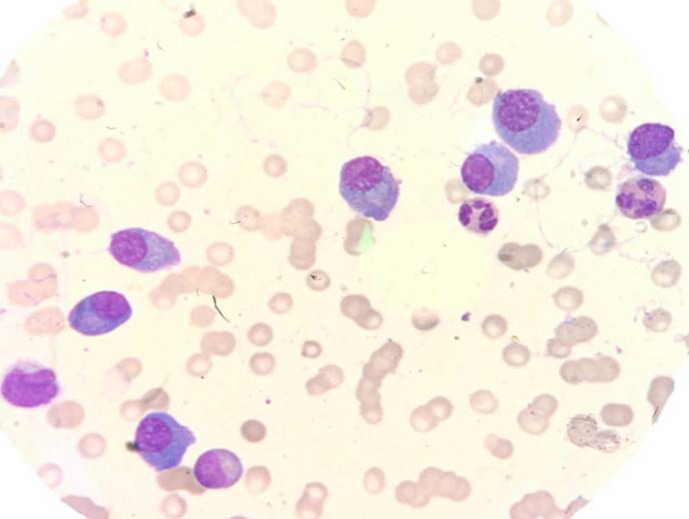
Aspiration smears show myeloma cells with mild atypia

**Figure 3:**
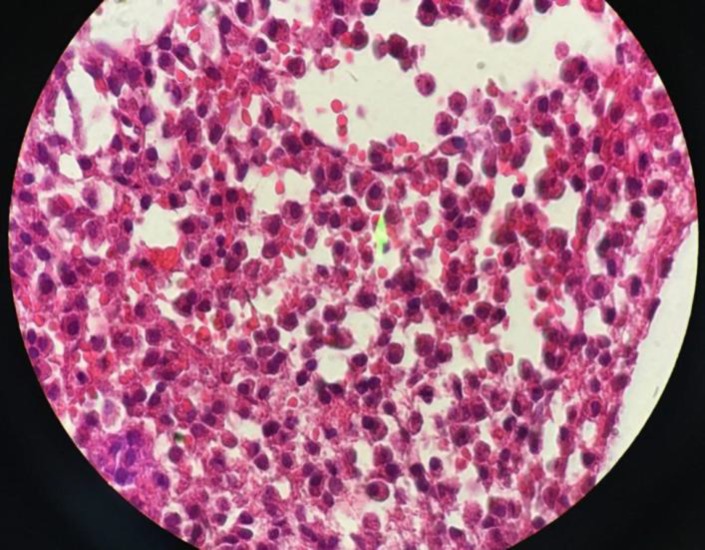
Trephine biopsy revealed large sheets of myeloma cells replace more than 90 percent of marrow space

Bone marrow aspiration and biopsy showed hyper cellular marrow with an approximate cellularity about 95% and diffuse infiltration of myeloma cells occupied more than 90% of marrow parenchyma (Figures 2, 3).

The diagnosis of multiple myeloma was made on the basis of bone marrow findings, serum protein electrophoresis, hypercalcemia and lytic lesions on skull x ray.

Intensive saline therapy, parenteral pamidronate, calcitonin and dexamethasone were initiated and patient was treated with bortezomib, cyclophosphamide and dexamethasone (VCD) regimen.

Intensive treatment of hypercalcemia and antimyeloma treatment reduced serum calcium level to 13 mg/dL. Continuation of treatment with corticosteroids, hydration and forced diuresis has had no effect in normalizing serum calcium level.

Reevaluation of hypercalcemia in eighth day revealed serum calcium 13 mg/dL, albumin 3 g/dL , phosphorous 2 mg/dL , Vit D_3_ 4 nmol/L level and iPTH that has been raised to 695 pg/ml. MIBI scanning with 99m Tc was negative for parathyroid adenoma.

At this time, serum ionized calcium level was measured and it was 3.9 mg/dL (NL range 4.4 – 5.3 mg/dL).

In our patient, hypercalcemia in the presence of normal or low serum ionized calcium denoted pseudohypercalcemia.

After diagnosis of pseudohypercalcemia, the patient discharged from hospital with prescription of calcium and Vit D.

After eight weeks of chemotherapy with VCD regimen as well as calcium and vitamin D supplementation, immunoglobin level decreased and serum calcium, albumin and PTH levels normalized 

## Discussion

 In severe hypercalemia marked symptoms such as polyuria, polydipsia, nausea, dehydration and

changes in consciousness are present^2^^,^^3^. Absence of associated symptoms in our patient indicates that ionized fraction is not increased. Several conditions are associated with pseudohypercalcemia, including prolonged use of tourniquet in sampling, dehydration, hyponatremia, excessive serum albumin, abnormally elevated calcium-binding globulin in hyper gammaglobulinemia and thrombocytosis ^4^^-^^7^^.^

Paraproteinemia can interfere with many biochemical laboratory measurement including glucose^8^, bilirubin^9^^,^^10^, sodium^11^^,^^12^, chloride^11^, calcium ^13^ and albumin ^14^.

Schwab et al. reported some cases of pseudohypercalcemia secondary to binding of calcium to immunoglobulins in patients suffering from multiple myeloma ^15^. Most of them had IgG

Myeloma with kappa light chains^15^.

In our patient, the persistent hypercalcemia and the subsequent elevated PTH level have led to the misdiagnosis of primary hyperparathyroidism concurrent with multiple myeloma. But, it seems that the increased PTH level was secondary to decreased ionized calcium level. Treatment of hypercalcemia and concomitant Vit D deficiency were the major causes of decreased ionized calcium. Normalization of PTH level after calcium and vitamin D supplementation indicates that our hypothesis regarding this patient laboratory abnormality is correct. 

## CONCLUSION

 In multiple myeloma patients with severe hypercalcemia, especially when signs and symptoms of hypercalcemia are absent, clinicians should recognize pseudohypercalcemia as an unusual cause to avoid unnecessary therapies. Measurement of ionized serum calcium is helpful in these situations
